# Effects of brief family psychoeducation on family caregiver burden of people with schizophrenia provided by psychiatric visiting nurses: a cluster randomised controlled trial

**DOI:** 10.1186/s12888-024-05884-z

**Published:** 2024-06-14

**Authors:** Naonori Yasuma, Sayaka Sato, Sosei Yamaguchi, Asami Matsunaga, Takuma Shiozawa, Hisateru Tachimori, Kazuhiro Watanabe, Kotaro Imamura, Daisuke Nishi, Chiyo Fujii, Norito Kawakami

**Affiliations:** 1 Ageonomori Clinic, Ageo, Saitama Japan; 2 Department of Community Mental Health and Law, National Institute of Mental Health, National Center of Neurology and Psychiatry, Kodaira, Tokyo Japan; 3 Department of Mental Health and Psychiatric Nursing, Tokyo Medical and Dental University, Bunkyo-ku, Tokyo, Japan; 4 Nursing Innovation Research Center, Graduate School of Health Care Sciences, Tokyo Medical and Dental University, Bunkyo-ku, Tokyo, Japan; 5 Department of Information Medicine, National Institute of Neuroscience, National Center of Neurology and Psychiatry, Kodaira, Tokyo Japan; 6 Department of Health Policy and Management, Keio University School of Medicine, Shinjuku-ku, Tokyo, Japan; 7 Department of Public Health, Kitasato University School of Medicine, Sagamihara, Japan; 8 Department of Digital Mental Health, Graduate School of Medicine, The University of Tokyo, Bunkyo-ku, Tokyo, Japan; 9 Department of Mental Health, Graduate School of Medicine, The University of Tokyo, Bunkyo-ku, Tokyo, Japan

**Keywords:** Caregiver burden, Schizophrenia, Brief family psychoeducation, Cluster randomised controlled trial

## Abstract

**Background:**

The purpose of this study was to examine the effects of a brief family psychoeducation (BFP) programme provided by psychiatric visiting nurses on caregiver burden of family caregivers of people with schizophrenia through a cluster randomised controlled trial (cRCT).

**Methods:**

The study was a two-arm, parallel-group cRCT. Forty-seven psychiatric visiting nurse agencies were randomly allocated to the BFP programme group (intervention group) or treatment as usual group (TAU; control group). Caregivers of people with schizophrenia were recruited by psychiatric visiting nurses using a randomly ordered list. The primary outcome was caregiver burden, measured using the Japanese version of the Zarit Burden Interview. Outcome assessments were conducted at baseline, 1-month follow-up, and 6-month follow-up. Intention-to-treat analysis was conducted to examine the effects of the BFP programme on caregiver burden.

**Results:**

Thirty-four psychiatric visiting nurse agencies and 83 family caregivers of people with schizophrenia participated in the study. The participant attrition rate was less than 20%. Adherence to the program was 100%. Compared with TAU group, the BFP programme group had decreased caregiver burden. However, this improvement was not significant at 1-month follow-up (adjusted mean difference [aMD] = 0.27, 95% CI = − 5.48 to 6.03, *p* = 0.93, *d* = 0.01) or 6-month follow-up (aMD = − 2.12, 95% CI = − 7.80 to 3.56, *p* = 0.45, *d* = 0.11).

**Conclusions:**

The BFP programme provided by psychiatric visiting nurses did not achieve significant decreases in caregiver burden. This result may be attributed to the difficulty in continuing the research due to the COVID-19 pandemic, which prevented us from achieving the targeted sample size necessary to meet the statistical power requirements, as well as to the participation of caregivers with relatively low burden. However, the program had the advantage of high adherence to treatment plan. Further studies should be conducted with a larger sample size and a more diverse sample that includes caregivers with a higher care burden.

**Trial registration:**

The study protocol was registered in the University Hospital Medical Information Network Clinical Trials Registry (UMIN000038044) on 2019/09/18.

## Background

Families caring for people with schizophrenia have many difficulties in their community lives [1]. They often do not acquire adequate general knowledge of schizophrenia [2]. Indeed, many family members have trouble coping with symptoms, communicating with people with mental illness, and keeping an appropriate distance from people with schizophrenia [2–4]. Due to these concerns, they feel both physical and psychological distress when taking care of people with schizophrenia and they wish to have their own free time [4]. Therefore, providing effective family interventions is an urgent matter in the field of community mental health services.

Previous studies have developed effective family interventions. Several systematic reviews showed that a family psychoeducation (FPE) programme is an effective approach that reduces caregiver burden [5, 6]. The FPE programme mainly includes components such as sharing information about the disorder, early warning signs, relapse prevention, as well as training in coping, communication, and problem-solving skills [7]. A FPE programme could improve caregivers’ knowledge about schizophrenia and related caregiving problems [8]. Improved knowledge could lead to a more positive appraisal of caregiving experiences as well as caregivers’ own self-efficacy, which decreases their burden [5].

Despite the accumulation of evidence, there are several barriers to FPE programme implementation. The implementation rate for FPE programmes at psychiatric facilities is low in Japan and other countries [9, 10]. A nationwide survey in Japan revealed that the implementation rate for FPE programmes at psychiatric facilities was 35.9% in hospitals and 14.5% in outpatient settings [9]. The initial report on the Schizophrenia Patient Outcomes Research Team’s treatment recommendations found that a FPE programme was provided to 31.6% of inpatients and 9.6% of outpatients [10]. One challenge in FPE programme implementation was the length of the intervention. A FPE programme usually ranges from 9 months to 2 years, which is impractical for medical staff, people with schizophrenia, and their families [11]. Other reasons include funding and staff shortages, as well as the need for training [12]. To address these issues, the development of a brief and implementable FPE programme within the existing mental health system was greatly needed [13].

The outcomes of brief family psychoeducation (BFP) programmes have been studied in caregivers of people with schizophrenia in previous studies. A BFP programme is defined as including five or fewer sessions or lasting no more than 3 months. It was easy to conduct for both practitioners and caregivers [14]. BFP programmes have been shown to significantly increase caregivers’ knowledge of the disorder, which could reduce relapse and rehospitalisation rates [15]. In addition, at least five randomised controlled trials (RCTs) showed that the BFP programme had an effect on caregiver burden [16–20] and three RCTs indicated a significant decrease in caregiver burden [17–19]. However, evidence of the effect of the BFP programme on caregiver burden has remained inconclusive due to at least two methodological problems. One is that the previous studies were single-centre RCTs, and contamination between the intervention and control groups might have occurred. The other is that the follow-up period was short, ranging from 1 to 3 months after the intervention. In other words, the long-term effects of the intervention remain unknown. Given the limitations of the previous studies, a cluster RCT (cRCT) with a longer follow-up period should be conducted.

Practical implementation strategies for the BFP programme in each health system need to be considered, in addition to a scientific evaluation of the effects. A BFP programme provided by psychiatric visiting nurses appears to be a potentially feasible and sustainable way of implementing the FPE programme in the Japanese clinical setting. Psychiatric visiting nurses routinely visit clients with schizophrenia and their family members. They have already built rapport with clients and family members and would be able to respond according to their needs, which means they could seamlessly provide a highly individualized BFP programme [21]. In addition, the system of psychiatric visiting nurses could easily be applied because the number of psychiatric visiting nurses has been increasing recently in Japan [22]. From a cost perspective, it would be possible to make family support a service that is reimbursable by the national health insurance to cover psychiatric visiting nurse consultancy fees. Taken together, incorporating the BFP programme into psychiatric visiting nurse practices could increase the implementation rate and lead to effective family interventions in the community setting in Japan.

### Hypothesis and aims

Given the above, the novelty of this study lies in using a long-term cRCT to elucidate the effects of BFP on caregiving burden, and in evaluating it within the context of psychiatric visiting nursing, an existing psychiatric healthcare system. Considering the low adoption rate of FPE, conducting an effectiveness validation of the easily implementable BFP in a real-world setting is deemed crucial. Additionally, given the simplicity of the BFP, it is expected to be of low intensity, and therefore, its effect size may be limited. There is a possibility that the program’s effects could be underestimated due to the exchange of information between psychiatric visiting nurses (program providers) in the intervention and control groups, or among the families (program recipients). To minimize this impact, the adoption of a cRCT offers advantages. Therefore, the aim of this study is to determine whether the caregiving burden among families caring for individuals with schizophrenia decreases when psychiatric visiting nurses provide BFP, through a 6-month follow-up cRCT. Based on the research aim, we hypothesised that a BFP programme provided by psychiatric visiting nurses could alleviate the burden on family caregivers of people with schizophrenia.

## Methods

### Trial design

A two-arm, parallel-group cRCT was performed. The study protocol [23] was registered in the University Hospital Medical Information Network (UMIN) Clinical Trials Registry (UMIN-CTR ID, UMIN000038044). First registration date was September 18, 2019. We reported study findings in accordance with the Consolidated Standards of Reporting Trials (CONSORT) for cRCTs [24].

### Setting and site selection at the cluster level

The first author (NY) approached 68 psychiatric visiting nurse agencies in 4 prefectures in Japan (Tokyo, Saitama, Kanagawa, and Chiba) managed by one organisation. Forty-seven visiting nurse agencies agreed to participate in the study.

The inclusion criterion for psychiatric visiting nurse agencies was that psychiatric visiting nurses provided services mostly to psychiatric patients rather than elderly people or those with physical diseases. In each agency, psychiatric visiting nurses must care for at least two people with schizophrenia who live with their family. There were no exclusion criteria at the cluster level.

### Randomisation at the cluster level

We randomly allocated psychiatric visiting nurse agencies into the BFP programme group (intervention group) or the treatment as usual (TAU) group. Randomization of clusters was performed prior to the collection of baseline data, and the results of the randomization were conveyed to each psychiatric visiting nurse agencies after the completion of participant recruitment. It was also stratified by the median of the average caseload of psychiatric visiting nurses in each agency because the service quality of a psychiatric visiting nurse is affected by caseload [25]. Family support is unlikely to be provided when a psychiatric visiting nurse has a high caseload. A researcher (HT) in the statistics department who was not involved in the study protocol development process created a random sequence table. Another researcher (SY) who was not involved in the intervention or analysis conducted the randomisation and informed each psychiatric visiting nurse agency of the randomisation results after the recruitment procedure at the individual level. The primary investigator (NY) was blinded through the entire randomisation process. Further information about the randomisation process was provided in the study protocol [23].

### Participant eligibility criteria and recruitment procedure at the individual level

The inclusion criteria for a family caregiver of a person with schizophrenia were as follows: (1) primary caregiver; (2) age over 20 years; (3) family member of a person with schizophrenia such as a parent, sibling, spouse, or child; and (4) living with the person with schizophrenia. There were no exclusion criteria for caregivers. The inclusion criteria for people with schizophrenia were as follows: (1) diagnosis of schizophrenia based on the International Statistical Classification of Diseases and Related Health Problems, 10th revision and (2) receiving services from psychiatric visiting nurses.

During the recruitment procedure at the individual level, we listed all potential participants (family caregivers of people with schizophrenia and people with schizophrenia) at each agency. Second, we created a randomly ordered list using a random number generator in the Stata statistical software program, version 15, in order to avoid selection bias at the individual level. Third, each psychiatric visiting nurse who attended a lecture on study design and ethical considerations recruited participants according to the randomly ordered list until five participants were recruited. Psychiatric visiting nurses also obtained written informed consent from people with schizophrenia and their caregivers. Only participants who voluntarily agreed to participate in the study were included.

The deadline for participant registration submitted to the ethics committee was October 31, 2020. In Japan, a state of emergency was declared after April 2020, which led to restrictions on the number of visits and duration of psychiatric visiting nurses’ visits from the perspective of infectious disease prevention. Consequently, conducting family psychoeducation, which requires longer visit times than usual, became a burden for both the participating facilities and the patients or their families, making further recruitment challenging.

### Intervention programme

This intervention program was a single-family intervention conducted by psychiatric visiting nurses that was based on the Family Intervention and Support in Schizophrenia: A Manual on Family Intervention for the Mental Health Professional [26]. Based on the concept of coproduction and patient and public involvement (PPI), we created this program through discussions and collaborations among members of the Family Association of Schizophrenia, psychiatric visiting nurses, FPE experts, psychiatrists, psychiatric nurses, clinical psychologists, and mental health social workers [27].

The program consisted of four sessions lasting 60 min each, which was completed over 1 month. Attendance of at least one session was required. In Session I, general knowledge about schizophrenia was covered, including its definition, causes, symptoms, and prognosis. The definition and causes were emphasized using the stress-vulnerability model and dopamine hypothesis, highlighting that schizophrenia is a brain disease that can affect anyone. It was important to explain biological causes, as some families believe that familial relationships are the cause of schizophrenia. Regarding symptoms, the session emphasized the challenges that individuals face in maintaining their typical way of life due to psychiatric symptoms. The course of the illness, including the prodromal, acute, and recovery phases, was explained, detailing the characteristics and management strategies for each phase. The prognosis discussed how schizophrenia is not necessarily a condition with a poor outcome. Medication therapy was addressed, acknowledging the difficulties of daily medication adherence, and discussing the necessity and safety of pharmacological treatment. The side effects of antipsychotic medications were explained using illustrations. The session concluded with an explanation of psychosocial treatments such as psychoeducation and daycare, and a knowledge check quiz was conducted. In Session II, various family concerns and problem-solving techniques were addressed. The session covered responses to hallucinations and delusions, signs of relapse, creating a crisis plan, managing worsening conditions, dealing with a family member staying indoors, reluctance to take medication, potential and occurring violence, and suspected self-harm or suicidal behaviors. Finally, problem-solving techniques were taught, and families practiced solving everyday caregiving issues using these methods. Session III dealt with engagement and communication training with the affected individual. It focused on understanding the patient’s feelings, Expressed Emotion (EE) theory, basic communication knowledge and skills, and methods to enhance resilience. The importance of showing understanding for the individual’s pessimistic views about their future and their difficulties was emphasized. EE theory also highlighted that it is understandable for families to exhibit high EE levels, and explained how changing the way families interact could potentially alter the individual’s symptoms and condition. Additionally, the session included practicing conversation using hypothetical case scenarios to think about better communication methods. Session IV focused on family recovery. It addressed the importance of family recovery, the significance of families living their own lives, considerations for physical and mental health, strategies for managing stress without exhaustion, experiences, and messages from members of a schizophrenia family association, and available community resources. This session emphasized the importance of both the individual and family having their own lifestyles and goals, encouraged families not to devote themselves solely to caregiving but to utilize various social resources to pursue their own lives. It also aimed to improve family members’ physical and mental health through knowledge of self-care and stress management. Experiences from three family association members were shared to help families understand that they are not alone in their struggles, aiming to alleviate their sadness and despair. Lastly, the session discussed community resources available and the importance of connecting with multiple supporters. We also described the development and contents in greater detail in the study protocol [23].

### Training and program adherence

The intervention team of psychiatric visiting nurses was provided with a 1-day group lecture before the intervention, which consisted of three parts. First, family caregivers of people with schizophrenia talked about their life problems and what they wanted psychiatric visiting nurses to do. Second, basic communication training was performed through role-playing. Third, the primary investigator (NY) equipped psychiatric visiting nurses with basic knowledge about FPE and explained the contents of this intervention tool and the points that the primary investigator wanted to emphasise.

With regard to program adherence, we created a checklist to confirm the date the programme was implemented and how many sessions psychiatric visiting nurses were actually able to conduct with participants. The checklist was self-reported by psychiatric visiting nurses at the end of each session.

### Treatment as usual (TAU) group

Family caregivers of people with schizophrenia in the control group received usual care from psychiatric visiting nurses. They did not receive any type of psychoeducation or supportive therapy.

### Outcomes

The following outcome measures were assessed at baseline prior to the intervention (T1), immediately after the completion of the intervention (1-month follow-up, T2), and 6 months after the baseline assessment (6-month follow-up, T3).

### Primary outcome for caregivers

#### Zarit burden interview (ZBI-22)

ZBI-22 was used for measuring caregiver burden. It consists of 22 items scored on a 5-point Likert scale from 0 (never) to 4 (nearly always) [28]. The total score ranges from 0 to 88, with higher scores indicating higher burden. Cut-off points are as following: 0–21, little or no burden; 21–40, mild to moderate burden; 41–60, moderate to severe burden; and 61–88, severe burden. The Japanese version of ZBI-22 has high test–retest reproducibility and internal consistency [29]. Construct validity has also been confirmed [30].

### Secondary outcome for caregivers

#### Kessler psychological distress scale (K6)

K6 was used to measure sub-clinical depression and anxiety disorders as part of a self-administered questionnaire, which consists of six items answered on a five-point Likert scale. Scores range from 0 to 24, with higher scores representing higher degrees of sub-clinical depression and anxiety disorder. The Japanese version has essentially equivalent screening performance as the original English version [30].

#### General self-efficacy scale (GSES)

GSES is a measure of self-efficacy in daily living, which includes 16 items with dichotomous questions [31]. In general, higher scores indicate better self-efficacy [31]. GSES has high test–retest reproducibility and internal consistency [31]. Construct validity has also been confirmed [31].

#### WHO-five well-being index (WHO-5)

WHO-5 was used to measure subjective well-being or quality of life based on positive mood (good spirits and relaxation), vitality (being active and waking up fresh and rested), and general interest (being interested in things). The scale consists of five items rated on a six-point Likert scale. Higher scores mean higher well-being. The Japanese version of WHO-5 has adequate internal consistency [32]. External concurrent validity and external discriminatory validity have also been confirmed in a previous study [32].

#### Knowledge of illness and drug inventory (KIDI)

KIDI was used to assess the knowledge regarding mental illness and the effects of medications on mental illness [33]. There were 2 sub-scales: 10 items assessing knowledge of mental illness and the remaining items assessing knowledge of the effects of antipsychotic drugs. This inventory consists of a self-reported inventory where respondents were asked to select the correct answer from three choices, with higher scores representing greater knowledge.

### Secondary outcomes in people with schizophrenia

#### Behaviour and symptom identification scale (BASIS-32)

BASIS-32 has been used to measure mental health. It includes 32 items on a 5-point Likert scale, where 0 indicates no difficulties and 4 indicates severe difficulties. The scale measured five factors: (1) relation to self and others (seven items); (2) depression/anxiety (six items); (3) everyday life and role functioning (nine items); (4) impulsive and addictive behaviour (six items); and (5) psychosis (four items). Internal consistency and construct validity of the Japanese version of BASIS-32 have been demonstrated [34]. For overall symptom severity, we used the average score of the 32 items.

### WHO-five well-being index (WHO-5)

WHO-5 was used to measure subjective quality of life based on positive mood (good spirits and relaxation), vitality (being active and waking up fresh and rested), and general interest (being interested in things). It consisted of five items rated on a six-point Likert scale. Higher scores mean higher well-being. The Japanese version of WHO-5 [32] had adequate internal consistency. It had been confirmed to have external concurrent validity and external discriminatory validity.

### Hospitalisation by 6-month follow-up

We created a self-reported measure for hospitalisation that included a dichotomous variable about whether a person with schizophrenia had been hospitalised during the past 6 months. The participating family caregiver reported this measure at the 6-month follow-up. If the person with schizophrenia had been hospitalised, the caregiver reported the date of admission.

### Sample size calculation

We calculated sample size according to the CONSORT guidelines for cRCTs [24], taking into account intra-class correlations (ICCs). The effect size of the BFP programme for individual caregiver burden was estimated based on a previous pre–post test [35]. The pre–post-test concluded that the standardised mean difference (*d*) of the BFP programme on caregiver burden was 0.46. Sample size was estimated as 76 in each arm based on an alpha error probability of 0.05 and power (1 − β) of 0.80, using G*Power version 3.1.9.2 [36, 37]. For cRCTs, this value should be multiplied by the design effect (1+[m − 1]ρ), where m is the average cluster size and ρ is the ICC [38]. The estimated ICC for the primary outcome in this study was set to 0.05 and the average number of caregivers per cluster was set at 5. Assuming an attrition rate of 20%, the required sample size is 110 caregivers in each arm. Thus, at least 44 visiting nurse agencies should be recruited.

### Statistical analyses

All analyses were conducted in accordance with intention to treat (ITT) model. The effect of the intervention on primary and secondary outcomes was estimated using linear mixed models, which allowed for missing data to be taken into account within the statistical model. In this study, a three-level model was used, with repeated measures nested in participants and participants nested in clusters. Time (baseline, 1-month follow-up, 6-month follow-up) was considered level 1, individual caregivers were considered level 2, and clusters (psychiatric visiting nurse agencies) were considered level 3. For fixed effects, condition (BFP programme versus TAU), time, and the two-way interaction effect of condition by time were included. Models were adjusted for baseline differences in caregiver socio-demographics such as age, gender, education, household income, family relationship to the person with schizophrenia, length of caregiving, and length of psychiatric visiting nurse system use. Subgroup analyses were conducted separately among respondents who had mild or higher caregiver burden (ZBI-22 score of 21 or higher) at baseline. A *p*-value of less than 0.05 was considered statistically significant. SPSS (Windows version 27) was used for statistical analysis.

### Changes to the protocol

Two changes for statistical analyses were made to the registered protocol [24]. One was analysis for rehospitalisation. We had intended to conduct Cox proportional hazards regression models with multiple levels for the dichotomous question of hospitalisation by 6-month follow-up. However, we were unable to perform the analysis because the date of admission was not available for one participant. Therefore, the number of hospitalisations by 6-month follow-up was just described and the chi-square test was conducted. The other change was a subgroup analysis to clarify the effect of the BFP programme based on the existence of caregiver burden (ZBI-22 score of 21 or higher). We determined that study participants had relatively a low level of caregiver burden compared with participants of previous studies [17–19] and the effect of the BFP programme may have been weakened by the floor effect.

## Results

### Participant flowchart

**Fig. 1:**
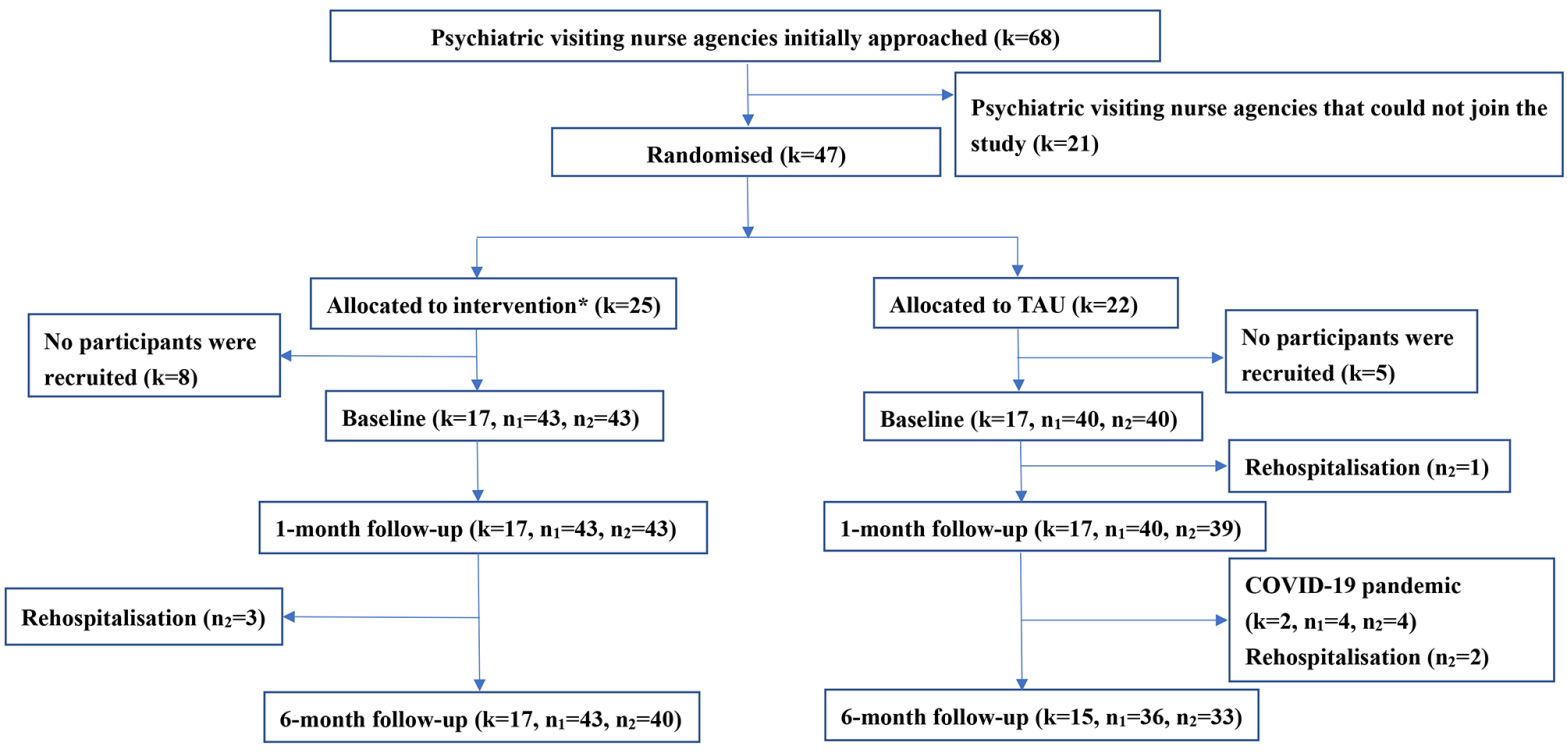
Study flow chart Randomisation results were revealed after the completion of recruitment k = number of psychiatric visiting nurse agencies, n_1_ = number of family caregivers of people with schizophrenia, n_2_ = number of people with schizophrenia. * Intervention group receive the brief family psychoeducation programme

Figure 1 shows the participant flowchart. Forty-seven psychiatric visiting nurse agencies (69%) agreed to participate in the study. After randomisation, 25 psychiatric visiting nurse agencies were allocated to intervention group and 22 psychiatric visiting nurse agencies were allocated to the TAU group. Thirteen psychiatric visiting nurse agencies (eight in the intervention group and five in the TAU group) could not recruit any participants. Of the 34 psychiatric visiting nurse agencies that recruited participants, 83 family caregivers of people with schizophrenia and 83 people with schizophrenia completed the baseline survey. At the 6-month follow-up survey, 17 agencies (100%), 43 family caregivers of people with schizophrenia (100%), and 40 people with schizophrenia (93%) in the intervention group and 15 agencies (88%), 36 family caregivers of people with schizophrenia (90%), and 33 people with schizophrenia (83%) in the TAU group completed the follow-up survey. Reasons for dropping out included rehospitalisation (n_2_ = 6) and the COVID-19 pandemic (k = 2, n_1_ = 4, n_2_ = 4), where k is the number of psychiatric visiting nurse agencies, n_1_ is the number of family caregivers of people with schizophrenia, and n_2_ is the number of people with schizophrenia.

### Baseline characteristics

Table 1 shows the demographic characteristics of family caregivers of people with schizophrenia (*n* = 83) at baseline. Most participating family caregivers of people with schizophrenia were elderly mothers who were not working. Half of participating family caregivers had experience with care for more than 10 years. Table 2 describes the demographic characteristics of people with schizophrenia (*n* = 83) at baseline. Most participating people with schizophrenia were unemployed and never married, had schizophrenia for more than 10 years, and had used psychiatric visiting nurse services for more than 3 years. The average number of lifetime hospitalisations was 3.0 in the intervention group and 4.7 in the TAU group. Three participants (7%) in the intervention group and two participants (5%) in the TAU group had been hospitalised within 6 months before the study.

**Table 1:** Demographic characteristics of family caregivers of people with schizophrenia (*n* = 83)

		Intervention (*n* = 43)	TAU (*n* = 40)
		*N* (%)	*N* (%)
**Age (mean ± SD)**		**69.4 ± 12.7**	**67.4 ± 12.3**
**Gender**	**Male**	**15 (34.9)**	**10 (25.0)**
	**Female**	**28 (65.1)**	**30 (75.0)**
**Education**	**Junior high school**	**9 (20.9)**	**2 (5.0)**
	**High school**	**17 (39.5)**	**22 (55.0)**
	**Some college**	**10 (23.3)**	**10 (25.0)**
	**University or higher**	**7 (16.3)**	**6 (15.0)**
**Employment**	**Yes**	**11 (25.6)**	**14 (35.0)**
**Household income**	**< 3 million yen**	**24 (55.8)**	**19 (47.5)**
	**< 5 million yen**	**5 (11.6)**	**10 (25.0)**
	**< 7.5 million yen**	**4 (9.3)**	**3 (7.5)**
	**≥ 7.5 million yen**	**2 (4.7)**	**3 (7.5)**
	**Unknown**	**8 (18.6)**	**5 (12.5)**
**Relationship to person with schizophrenia**	**Parent**	**33 (76.7)**	**33 (82.5)**
	**Spouse**	**6 (14.0)**	**2 (5.0)**
	**Sibling**	**1 (2.3)**	**2 (5.0)**
	**Child**	**1 (2.3)**	**3 (7.5)**
	**Other**	**2 (4.7)**	**0 (0)**
**Care time**	**<5 years**	**13 (30.2)**	**7 (17.5)**
	**<10 years**	**7 (16.3)**	**8 (20.0)**
	**<15 years**	**6 (14.0)**	**5 (12.5)**
	**<20 years**	**3 (7.0)**	**6 (15.0)**
	**≥ 20 years**	**14 (32.6)**	**14 (35.0)**

**Table 2:** Demographic characteristics of people with schizophrenia (*n* = 83)

		Intervention (*n* = 43)	TAU (*n* = 40)
		*N* (%)	*N* (%)
**Age (mean ± SD)**		**45.1 ± 14.5**	**42.7 ± 9.9**
**Gender**	**Male**	**19 (44.2)**	**18 (45.0)**
	**Female**	**24 (55.8)**	**22 (55.0)**
**Education**	**Junior high school**	**11 (25.6)**	**11 (27.5)**
	**High school**	**20 (46.5)**	**18 (45.0)**
	**Some college**	**5 (11.6)**	**5 (12.5)**
	**University or higher**	**7 (16.3)**	**6 (15.0)**
**Employed**	**Yes**	**9 (20.9)**	**5 (12.5)**
	**No**	**34 (79.1)**	**35 (87.5)**
**Marital status**	**Never married**	**33 (76.7)**	**35 (87.5)**
	**Married**	**7 (16.3)**	**2 (5.0)**
	**Divorced**	**3 (7.0)**	**3 (7.5)**
**Duration of schizophrenia**	**<5 years**	**12 (27.9)**	**8 (20.0)**
	**<10 years**	**9 (18.6)**	**8 (22.5)**
	**<15 years**	**6 (14.0)**	**4 (10.0)**
	**<20 years**	**3 (7.0)**	**5 (12.5)**
	**≥ 20 years**	**14 (32.6)**	**14 (35.0)**
**Duration of using psychiatric visiting nurse services**	**<1 year**	**4 (9.3)**	**7 (17.5)**
	**<3 years**	**19 (44.2)**	**13 (32.5)**
	**≥ 3 years**	**20 (46.5)**	**20 (50.0)**
**Number of lifetime hospitalisations (mean ± SD)**		**3.0 ± 2.7**	**4.3 ± 5.0**
**Hospitalisation within 6 months before the study**	**Yes**	**3 (7.0)**	**2 (5.0)**

### Effects of the BFP programme on the primary outcome

Table 3 presents the effects of the BFP programme by psychiatric visiting nurses on the primary outcome. The BFP programme decreased caregiver burden but the effect size was small. However, adjusted mean differences (aMDs) between the groups were not significant at both the 1-month follow-up (aMD = 0.27, 95% CI = − 5.48 to 6.03, *p* = 0.93, *d* = 0.01) and the 6-month follow-up (aMD = − 2.12, 95% CI = − 7.80 to 3.56, *p* = 0.45, *d* = 0.11). Table 4 shows the subgroup analysis. The effect size was larger when stratified by the presence of care burden (ZBI-22 score of 21 or higher) compared to the whole sample, but there were no significant differences between the groups.

**Table 3:** Effect of the intervention on primary and secondary outcomes at baseline (T1), 1-month follow-up (T2), and 6-month follow-up (T3)

		Intervention			TAU			Adjusted mean difference (95% CI)	*p*	Effect size (Cohen’s d)
**Caregivers of people with schizophrenia**		***n***	**M**	**SD**	***n***	**M**	**SD**			
**Primary outcome**										
**ZBI-22**	**T1**	**43**	**33.0**	**19.6**	**40**	**29.9**	**18.2**			
	**T2**	**42**	**29.2**	**17.9**	**40**	**25.9**	**17.1**	**0.27 (-5.48 to 6.03)**	**0.93**	**0.01**
	**T3**	**42**	**28.8**	**16.0**	**35**	**26.6**	**16.5**	**-2.12 (-7.80 to 3.56)**	**0.46**	**0.11**
**Secondary outcome**										
**K6**	**T1**	**43**	**5.7**	**5.8**	**40**	**5.5**	**4.6**			
	**T2**	**43**	**6.1**	**4.6**	**40**	**5.2**	**4.3**	**0.60 (-1.14 to 2.33)**	**0.49**	**0.11**
	**T3**	**42**	**6.2**	**4.6**	**35**	**6.1**	**4.5**	**-0.32 (-2.12 to 1.48)**	**0.72**	**0.06**
**GSES**	**T1**	**43**	**8.7**	**3.5**	**36**	**8.9**	**4.7**			
	**T2**	**42**	**9.0**	**4.0**	**39**	**9.1**	**5.2**	**-0.19 (-1.51 to 1.14)**	**0.78**	**0.05**
	**T3**	**41**	**8.5**	**4.2**	**35**	**8.8**	**5.1**	**-0.16 (-1.46 to 1.13)**	**0.80**	**0.04**
**WHO-5**	**T1**	**43**	**13.7**	**5.5**	**40**	**14.1**	**5.7**			
	**T2**	**43**	**13.3**	**5.6**	**40**	**13.9**	**5.9**	**-0.12 (-2.22 to 1.97)**	**0.91**	**0.02**
	**T3**	**42**	**13.1**	**4.8**	**35**	**13.5**	**5.9**	**0.28 (-1.76 to 2.32)**	**0.78**	**0.05**
**KIDI**	**T1**	**43**	**14.4**	**3.6**	**40**	**14.7**	**3.3**			
	**T2**	**42**	**15.9**	**3.1**	**37**	**15.6**	**3.0**	**0.79 (-0.28 to 1.87)**	**0.15**	**0.23**
	**T3**	**41**	**16.1**	**3.1**	**35**	**15.3**	**3.2**	**1.13 (-0.09 to 2.34)**	**0.068**	**0.33**
**People with schizophrenia**										
**Secondary outcome**										
**BASIS-32**	**T1**	**43**	**0.84**	**0.74**	**39**	**0.99**	**0.70**			
	**T2**	**43**	**0.77**	**0.67**	**39**	**0.79**	**0.65**	**0.11 (-0.16 to 0.38)**	**0.42**	**0.15**
	**T3**	**37**	**0.75**	**0.54**	**32**	**0.92**	**0.47**	**-0.06 (-0.31 to 0.19)**	**0.61**	**0.08**
**WHO-5**	**T1**	**42**	**13.5**	**6.3**	**39**	**12.1**	**6.7**			
	**T2**	**42**	**13.8**	**6.5**	**38**	**12.7**	**6.3**	**-0.58 (-2.77 to 1.61)**	**0.60**	**0.09**
	**T3**	**39**	**13.9**	**6.1**	**33**	**12.8**	**5.8**	**-0.11 (-2.35 to 2.13)**	**0.93**	**0.02**

**Table 4:** Subgroup analysis at baseline (T1), 1-month follow-up (T2), and 6-month follow-up (T3)

		Intervention			TAU			Adjusted mean difference (95% CI)	*p*	Effect size (Cohen’s d)
		***n***	**M**	**SD**	***n***	**M**	**SD**			
**ZBI-22 score ≥ 21**	**T1**	**28**	**44.6**	**13.4**	**25**	**39.6**	**16.0**			
	**T2**	**27**	**35.7**	**17.9**	**25**	**34.7**	**15.1**	**-3.86 (-11.4 to 3.7)**	**0.31**	**0.26**
	**T3**	**28**	**35.5**	**14.4**	**21**	**34.5**	**15.7**	**-4.44 ( -11.8 to 2.9)**	**0.23**	**0.30**

### Effects of the BFP programme on secondary outcomes

Table 3 shows the effects of the BFP programme on secondary outcomes. For family caregivers of people with schizophrenia, there were no statistically significant effects of the BFP programme on K6, GSES, WHO-5, or KIDI at the 1-month follow-up. No statistically significant effects of the BFP programme were observed for K6, GSES, or WHO-5 at the 6-month follow-up. For people with schizophrenia, BASIS-32 and WHO-5 scores were not statistically significantly different between the groups at the 1-month or 6-month follow-up. The number of people with schizophrenia who were admitted to the hospital by 6-month follow-up was five (12%) in the intervention group and four (10%) in the TAU group (χ^2^ = 0.06, *p* = 0.81).

### Program adherence

All participants (100%) in the intervention group completed the four sessions of the BFP programme in accordance with the protocol.

## Discussion

Contrary to our hypothesis, we found that the BFP programme did not have a significant effect on caregiver burden among family caregivers of people with schizophrenia compared with the TAU group. There were no significant differences in secondary outcomes. However, the attrition rates of participants were 7% in the intervention group and 13.7% in the control group through the final follow-up, and adherence to the program was 100% throughout the intervention period.

The BFP programme provided by psychiatric visiting nurses had no significant effect on caregiver burden. Our results were consistent with the study of Shinde et al. [16] and Shiraishi et al. [20]. The most plausible reason for the absence of significant differences in both the primary and secondary outcomes is that the sample size in this study was smaller than planned. This resulted in limited statistical power, which may have affected the results. Original research protocol planned to continue recruitment of participants to meet the sample size. On the other hand, the COVID-19 pandemic adversely influenced the research activities. For example, informed consent interview and the 60-minute face-to-face BFP sessions increased the risk of infection, making additional recruitment and intervention implementation difficult. In addition, the COVID-19 pandemic reduced the frequency of visits and shortened the time per visit in the usual support. This resulted in difficulty to ensure time spent on BFP. In summary, small sample size and limited usual services due to the COIVD-19 pandemic may affect the results that we did not find significant effects of the BFP program. Additionally, there were five assumptions that can be considered for the lack of significant differences. First is another potential influence of the COVID-19 pandemic. The 6-month follow-up survey was conducted during the COVID-19 pandemic lockdowns in Japan. There might have been unusual caregiver burden that was difficult to alleviate through intervention [39]. In other words, the COVID-19 and lockdowns may adversely affect the effectiveness of the BFP program. Second, the caregivers who participated in this study had relatively mild to moderate (21 ≤ ZBI-22 score ≤ 40) caregiver burden, which could have led to the floor effect and weakened the effect of the BFP programme on caregiver burden. This was supported by the studies of Sharif et al. [17], Khoshknab et al. [18], and Hasan et al. [19], which targeted populations with a high caregiver burden and had significant results, while the study of Shiraishi et al. [20] included populations with a low caregiver burden and did not have significant results. Our subgroup analysis also supported the fact that the effect size was larger when stratified by level of care burden. Third, a Japanese culture might be related to the results. In a culture of shame in which the Japanese are uniquely concerned about public views, the participating caregivers may have under-reported their burden. In addition, Japanese families have a long history of being forced to take on caregiving responsibilities [40] and tend to prioritise improving the lives of people with schizophrenia over their own internal challenges [39]. As a results, some family members tend to feel ashamed of having a sense of caregiving burden. This may have contributed to the floor effect and weakened the effect of intervention. Forth, the intervention might have been inadequately implemented. Although psychiatric visiting nurses received a 1-day lecture, they might not have been able to acquire all the skills needed to successfully provide the BFP programme. Fifth, the psychiatric visiting nurses in the control group might have become more aware of family support because of participating in the study. This may have led to more extensive normal family support and a reduction in caregiver burden.

This study had four strengths. First, this study had a cRCT design and included 34 psychiatric visiting nurse agencies in 4 prefectures in Japan (Tokyo, Saitama, Kanagawa, and Chiba). The multi-site implementation could have increased the representativeness of the study population seen in the real world. Second, the attrition rate of participants was less than 20% from the baseline to the 6-month follow-up, which could have reduced withdrawal bias. Third, all participants in the BFP group attended all the sessions, which could imply that family caregivers and people with schizophrenia considered the programme meaningful, although we did not assess the usefulness of the programme through qualitative interviews. Fourth, the BFP programme contents were developed through a co-productive process that involved a wide variety of stakeholders, including family members and experts, suggesting that the programme could be improved and enriched by adding perspectives based on the unique experiences of family caregivers that are difficult for experts to recognize.

This study had three limitations. First, the number of participants was small compared to the planned sample size, and statistical power was limited. Second, the characteristics of participants might have been different from those of non-participants such as caregivers with high care burden, which might have reduced the external validity of this study. Third, there was the possibility that psychiatric visiting nurses in the TAU group could have received information about the BFP programme from psychiatric visiting nurses in the intervention group because the nurses in the intervention and TAU groups work for the same organisation. This possible contamination might have weakened the intervention effect.

### Implications for future research

This study yields several implications for future research endeavours. Firstly, future studies should undertake a cRCT with adequate sample sizes, focusing on enrolling families enduring high caregiving burdens. Refinements in recruitment methodologies are needed to ensure that families receive comprehensive information about the study through their accessible means, such as an online session. Moreover, tailoring the intervention schedule to offer more flexibility for families with heavy caregiving burdens could enhance participation. For instance, adjusting the duration based on the needs of the caregivers might be beneficial. This is because some caregivers may find shorter, more focused sessions to be helpful, while others may prefer extended interactions for deeper discussions. Providing separate, private consultations for family members, distinct from those with schizophrenia, might be a beneficial strategy, given that caregivers could have reservations or concerns that they are reluctant to disclose in a shared setting. Indeed, the COVID-19 pandemic significantly impacted the implementation of our study. The challenges presented by the pandemic highlight the need for online family psychoeducation. This could be a crucial tool in ensuring continuous support and effective interventions in similar future scenarios. A recruitment strategy that screens families based on caregiving burden could potentially increase participation rates. Secondly, the BFP could benefit from qualitative surveys targeting various families and psychiatric visiting nurses to accumulate feedback on satisfaction and potential enhancements. Such an approach would ensure that the program reflects a variety of perspectives, thereby improving its generalizability. Finally, psychiatric visiting nurses’ quality evaluation and reproducibility of the BFP are essential. The development of a fidelity scale could facilitate the assessment of the reproducibility and consistency of the BFP across diverse settings. Essential advancements toward enhancing the program’s efficacy might also encompass evaluating practical training for psychiatric visiting nurses, integrating expert insights into family support training, and examining the resulting influence of such training.

## Conclusions

In the present study, BFP provided by psychiatric visiting nurses did not significantly reduce the family’s sense of caregiving burden. Therefore, it is difficult to recommend the BFP programme developed by this study for use in clinical practice as is. On the other hand, the results, in which all study participants in the BFP intervention group attended all sessions, suggest that BFP delivered by psychiatric visiting nurses is partly feasible. Therefore, future research is expected to develop and study more effective practices while maintaining feasibility. Specific challenges for future research will require cRCTs to be conducted with study subjects that meet the sample size and include family members with higher care burden. At the same time, it is necessary to improve the BFP programme and ensure the quality-of-service provision by creating fidelity, and to reconsider the content of training for psychiatric visiting nurses and verify its effectiveness.

## Data Availability

The data that support the findings of this study are available from the corresponding author upon request. The data are not publicly available due to privacy and ethical restrictions. Participants were not informed about public data access during the informed consent process. When we receive a reasonable request for data, the data will be made upon approval by the Research Ethics Committee of the Graduate School of Medicine and the Faculty of Medicine at The University of Tokyo, Japan.

## Abbreviations

FPE: family psychoeducation
BFP: brief FPE
BAS: Burden Assessment Scale
RCT: randomised controlled trial
J-ZBI_8: Zarit Burden Interview, short version
cRCT: cluster randomised controlled trial
UMIN: University Hospital Medical Information Network
CONSORT: Consolidated Standards of Reporting Trials
TAU: treatment as usual
PPI: patient and public involvement
ZBI-22: Zarit Burden Interview
K6: Kessler Psychological Distress Scale
GSES: General Self-Efficacy Scale
WHO-5: WHO-Five Well-being Index
KIDI: Knowledge of Illness and Drug Inventory
BASIS-32: Behavior and Symptom Identification Scale
ICC: intra-class correlation
ITT: intention to treat
COVID-19: Coronavirus disease 2019
